# Epidemiology of talus fractures in Finland: a nationwide register study from 1997 to 2020

**DOI:** 10.1186/s12891-024-08141-2

**Published:** 2024-12-19

**Authors:** Evgenii Brushtein, Nikke Partio, Tuomas T. Huttunen, Jussi P. Repo, Heikki Mäenpää, Ville M. Mattila

**Affiliations:** 1 Unit of Musculoskeletal Surgery, Department of Orthopedics and Traumatology, Tampere University Hospital, Tampere, Finland; 2 Tampere University Heart Hospital, Tampere University Hospital, Tampere, Finland; 3 Unit of Musculoskeletal Surgery, Department of Orthopedics and Traumatology, Tampere University Hospital, Kuntokatu 2, Tampere, 33520 Finland

**Keywords:** Talus, Fracture, Incidence, Operations

## Abstract

**Background:**

The aim of this study was to estimate the nationwide incidence of talus fractures (per 100 000 person-years) and to determine the incidence of operative treatment in the Finnish population.

**Methods:**

Based on Finnish Care Register for Health Care data, all patients 18 years and older admitted to hospital with talar fractures between 1997 and 2020 were included.

**Results:**

During the 24-year study period, 5247 patients with primary or secondary diagnoses of talar fracture were identified. The mean incidence during the whole study period was 50.4 per 100 000 person-years. There was a clear increase starting from the year 2009 (61.4 per 100 000 person-years), that continued and the incidence in 2018 raised up to 106.8 person-years. The total incidence of operations performed on talar fractures remained almost the same during the study period (9.1 per 100 000 person-years from 1997 to 2020).

**Conclusions:**

While the incidence of performed operations had remained stable, the incidence of talar fractures in Finland has increased during the last decades. The increase was more prominent in men. The observed change is possibly due to the progress and availability of computer tomography.

**Level of Evidence:**

III

## Introduction

Fractures of the talus are rather rare injuries, accounting for 0.3–1% of all fractures in the human body and 3–6% of fractures in the foot [1–3]. The spectrum of talar fractures ranges from small avulsion fractures or exactly positioned fractures to severe injuries with dislocation, subluxation, or fragmentation of bone.

The treatment of talus fractures is strongly based on the anatomical location of the fracture, displacement, classification, and joint alignment [4]. The aim of operative treatment is to reduce joint displacement and malalignment and often requires open reduction and internal fixation (ORIF) with plates, metal or bioabsorbable screws [5].

In the present study, we aim to investigate the incidence of talus fractures in Finland and determine the trends in operative treatment between 1997 and 2020.

## Methods

To better understand trends in the hospitalization and surgical treatment of patients with talar fractures, the Care Register for Health Care was reviewed between the years 1997 and 2020. All patients aged 18 years or older who were admitted to hospitals with talar fracture were included.

Founded in 1967, the Care Register for Health Care is an excellent database for epidemiologic studies, as it contains data on age, sex, domicile of the patient, length of hospital stay, primary and secondary diagnoses, and operations performed during the hospital stay. The data collected by the CRHC are mandatory for all private and public hospitals and other health care institutions. The validity of the CRHC has been found to be excellent regarding both the coverage and the accuracy of the database [6–10]. The limitation of the study is that the Finnish CRHC does not provide the laterality of the fracture. This created a challenge to differentiate between follow-up visits, re-operations on the index talus, new operations and fractures of the contralateral talus. Fortunately, bilateral talar fractures are very rare and only a few bilateral talar fractures have been reported to date [11].

The main outcome of the study was the number of patients hospitalized due to talar fracture as a primary or secondary diagnosis (ICD-10 code S92.1 between 1997 and 2020). The ICD-10 coding system has been used in Finland since 1996, and the included operative treatment codes were NHJ50, NHJ99, and NHJ10 for open reduction and internal fixation (ORIF). Data from the whole study period (1997 to 2020) were pooled for analysis (ORIF or conservative treatment). If a person had multiple hospitalizations with a talar fracture diagnosis, only the first period of hospitalization was included in the analysis.

### Statistical analysis

To compute the incidence rates of talar fractures leading to hospitalization, the annual midyear populations were obtained from Official Statistics of Finland, a statutory, electronic population register. The incidence of talar fractures and the incidence of operative treatment (per 100 000 person-years) were based on the results of the entire adult population of Finland rather than cohort-based estimates. Therefore, 95% confidence intervals were not calculated. Statistical analysis was performed using PASW19.01. According to Finnish legislation, ethical approval for this study was not required due to the register-based design of the study. Data acquisition approval was obtained from the Finnish Institute for Health and Welfare (Dnro: THL/1800/5.05.00/2019).

## Results

During the 24-year study period, a total of 5247 patients were hospitalized in Finland with a primary or secondary diagnosis of talar fracture. Of the 5247 patients hospitalized with talar fracture, the majority (69.6%) were men (*n* = 3653) and 30.4% were women (*n* = 1594). The mean incidence during the whole study period was 50.4 per 100 000 person-years.

**Fig. 1:**
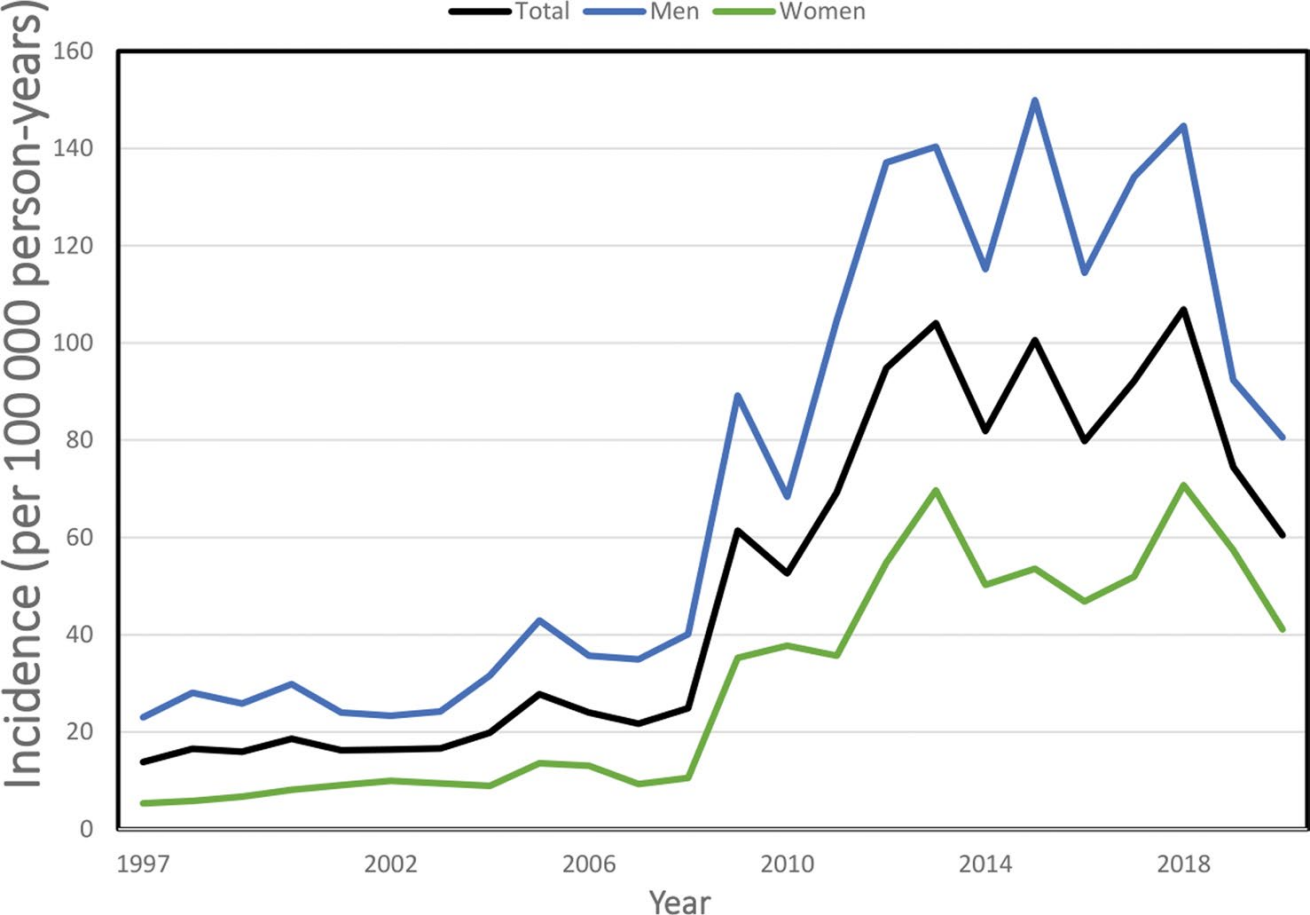
Incidence of talar fractures in Finland between 1997 and 2020

We observed a clear increase in the incidence of talar fracture starting from the year 2009. In 1997, the incidence was 13.8 per 100 000 person-years (23.0 per 100 000 person-years in men and 5.3 in women), which subsequently increased to 24.8 per 100 000 person-years in 2008 (40.0 per 100 000 person-years in men and 10.6 in women). Furthermore, this trend continued and the mean incidence in 2018 was 106.8 per 100 000 person-years (Fig. 1).

**Fig. 2:**
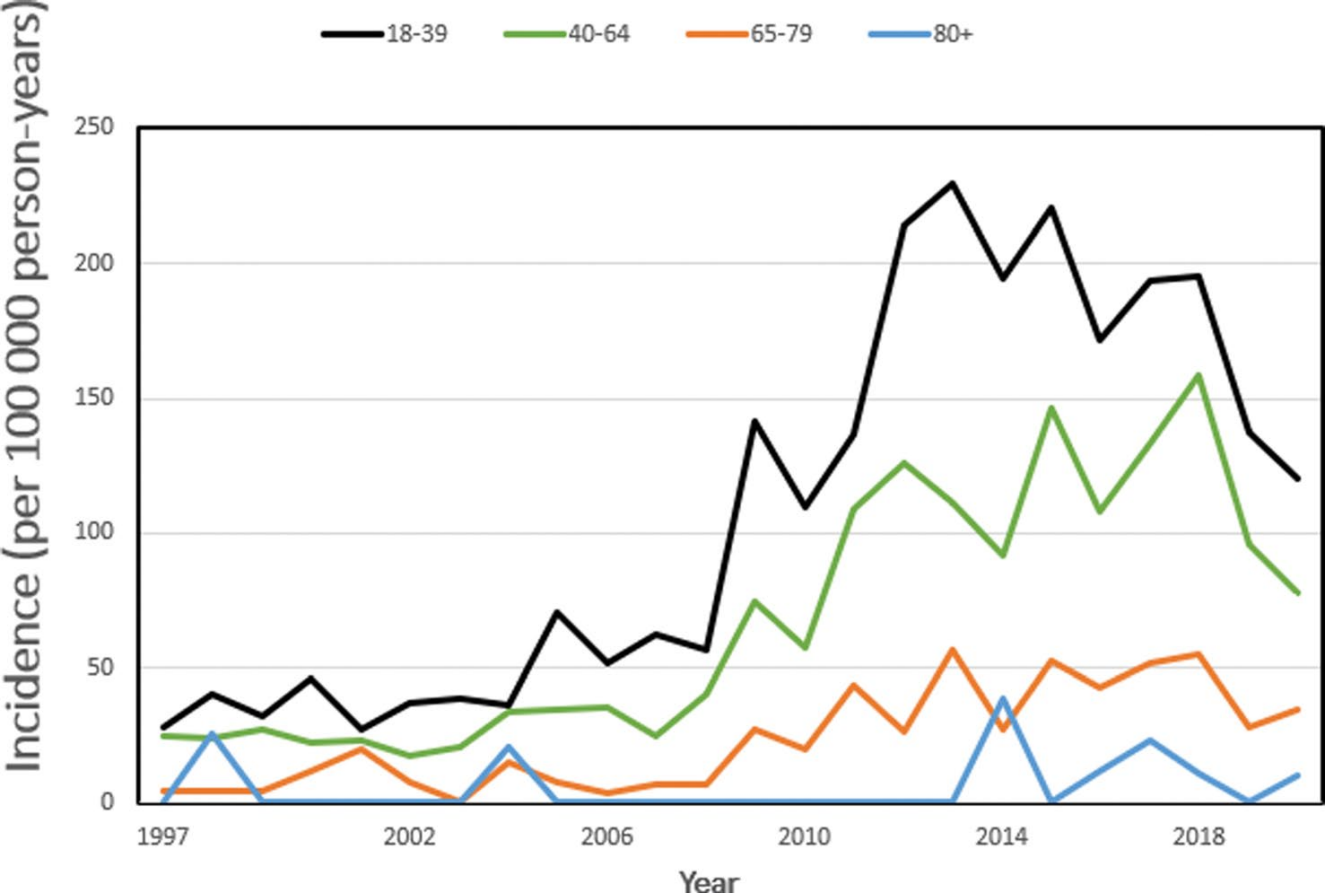
Incidence of talar fractures in Finland between 1997 and 2020 in men by age

**Fig. 3:**
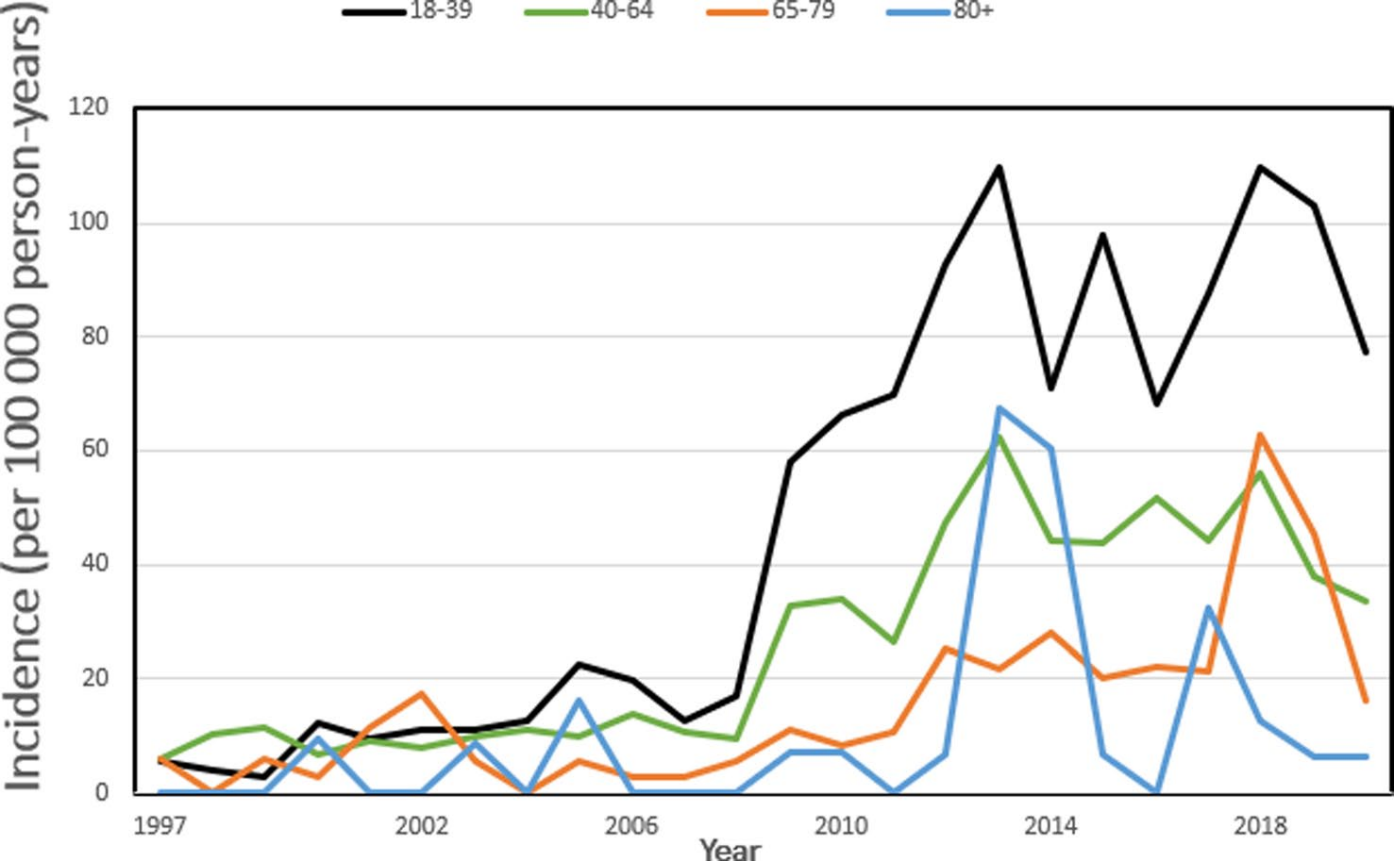
“Incidence of talar fractures in Finland between 1997 and 2020 in women by age

The age and sex-specific analysis of the whole study period revealed, that’s the highest incidence was seen in men in the 18 to 39 age group (the incidence rate was 107.9 per 100 000 person-years). Women aged 80 and older represented the only group with a higher incidence of talar fractures in women compared to men, where the mean incidence in women was 10.3 per 100 000 person-years vs. 5.9 per 100 000 person-years in men. Figures 2 and 3.

Isolated talar fracture was found in 1553 patients (29.6%), while others (*n* = 3694) had additional diagnoses in the CRHC. The most common concomitant fractures occurred in the calcaneus (11.2%, *n* = 413), the distal tibia (6.1%, *n* = 227), the metatarsal bones (5.6%, *n* = 206), and the medial malleolus (5.3%, *n* = 198), followed by bimalleolar/trimalleolar fractures (5.1%, *n* = 190).

**Fig. 4:**
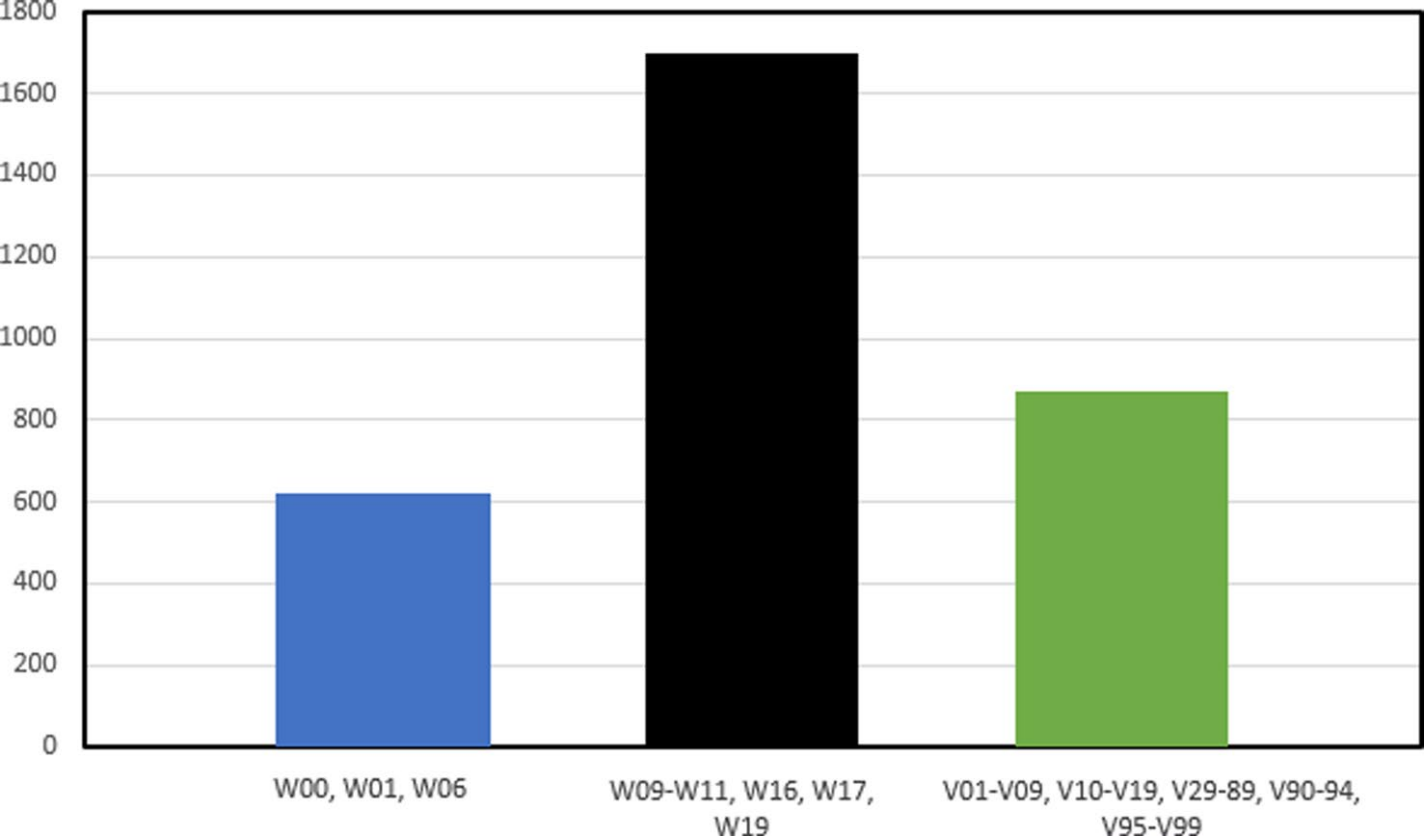
Most frequent mechanisms of injury of talar fractures in Finland between 1997 and 2020

The most frequent trauma mechanism was high-energy fall, which means falling from over than 2 floors or more than 6 m high (53.2%, ICD-10 codes W09-W11, W16, W17, W19), followed by traffic accident (27.2%, ICD-10 codes V01-V09, V10-V19, V29-V89, V90-V94, V95-V99) and fall on the same level (19.5%, ICD-10 codes W00, W01, W06). Figure 4; Table 1.

**Table 1:** List of diagnosis and operation codes

Procedure code	Explanation
NHJ50	Open reduction of fracture of foot or toe
NHJ99	Other fracture surgery of foot or toe
NHJ10	Internal fixation of fracture of ankle using wire, rod, cerclage or pin
Diagnosis Code	Explanation
S92.1	Talus fracture
External Cause Code	Explanation
W00	Falling on icy or snowy conditions
W01	Falling on the same level
W06	Falling from the bed
W19	Unspecified fall or drop
V01-V09	Pedestrian traffic accidents
V10-V19	Cycling accidents
V29-V89	Other land traffic accidents
V90-V94	Water traffic accidents
V95-V97	Accidents in the air and space
V98-99	Other or unspecified traffic accidents
W09	Falling or tumbling off playground equipment
W10	Falling or tumbling on or from stairs
W11	Falling on or from a ladder
W16	Diving or jumping into water resulting in an injury other than drowning
W17	Falling

**Fig. 5:**
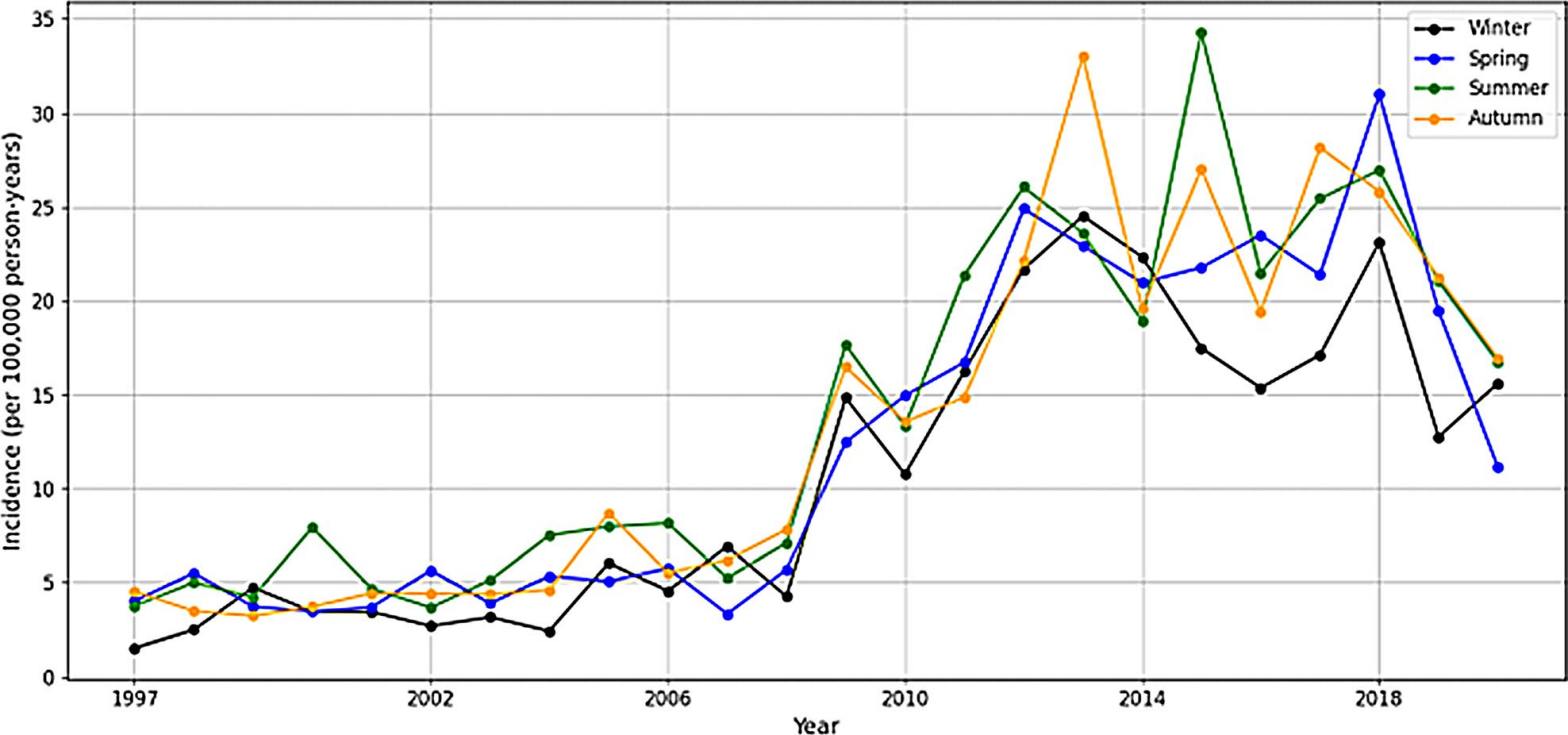
Seasonable variations in the incidence of talar fractures in Finland between 1997 and 2020

The study also reveals a seasonal variation in the incidence of talar fractures, with a notable increase observed across all seasons, particularly summer (of peak incidence 34.3 in the year 2015 and 21.4 in the year 2011) and autumn (with the peak incidence of 33.0 in the year 2013 and 27.0 in the year 2015). Winter and spring also showed increases in certain years, with a higher incidence period in the year 2018 for winter seasons (23.1 per 100 000 person-years) and in the year 2018 for spring seasons (30.1 per 100 000 person-years). Figure 5.

Median length of hospital stay was 1 day for conservatively treated talar fractures and 5 days for operatively treated fractures (range, 1–83 days).

**Fig. 6:**
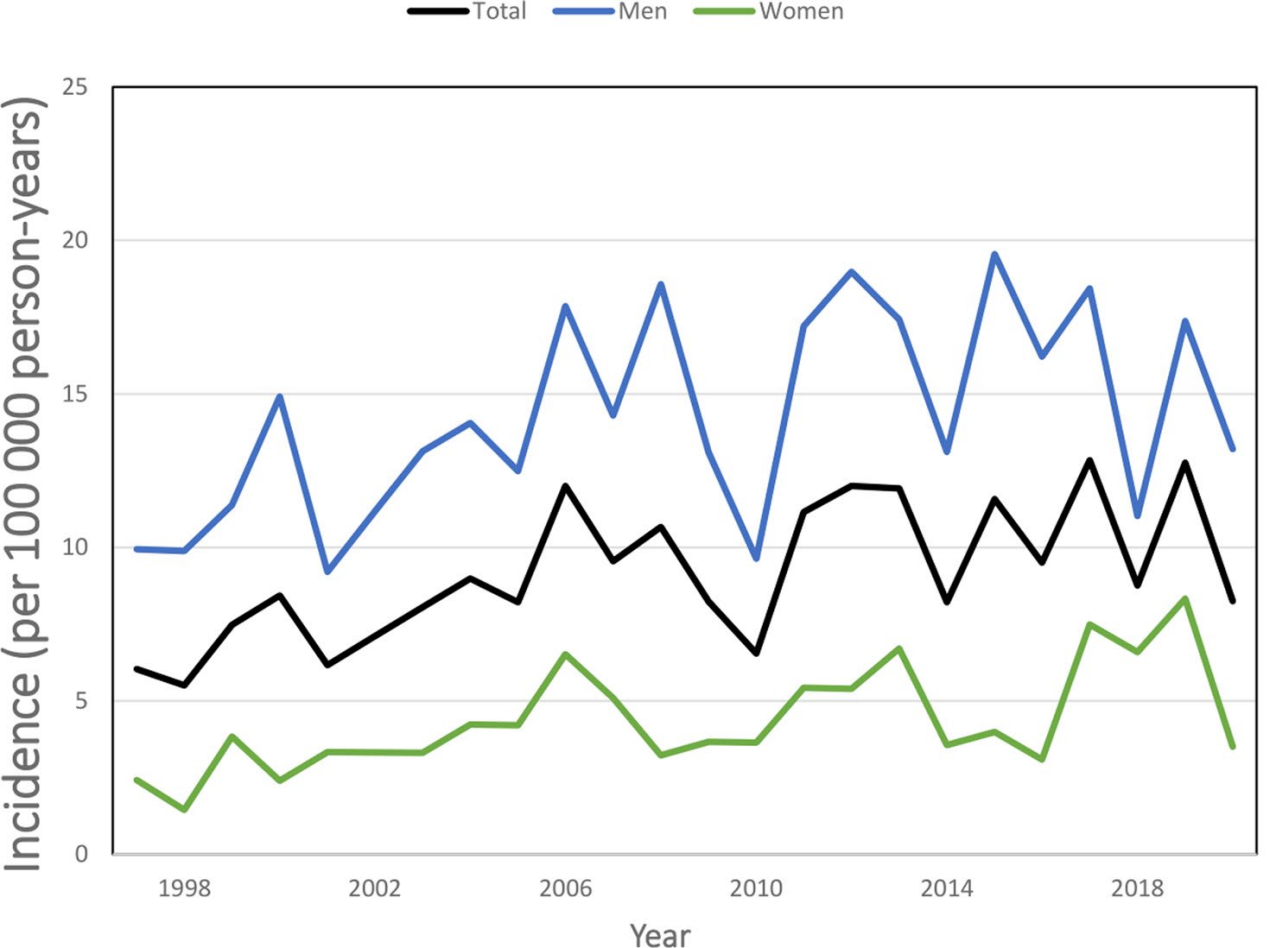
Incidence of operatively treated fractures

The incidence of operatively treated patients is shown in Fig. 6 (937 (27.6%) fractures were operated).

The total incidence of operations performed on talar fractures increased during the last decade of the study by 24.7% (8.1 per 100 000 person-years from 1997 to 2008 and 10.1 per 100 000 person-years during the years 2009 to 2020).

## Discussion

Our study described the incidence and trends of talar fractures and their operative treatment in Finland between 1997 and 2020. Although we found an increased incidence rate of talar fracture, the incidence of operatively treated talar fractures did not change markedly.

In the present study, the nationwide incidence of talar fractures during the first 10 years was at a steady level of 19.3 per 100 000 person-years. However, in 2008, the incidence began to increase sharply and rose to 81.5 per 100 000 person-years between 2009 and 2020. To our knowledge, no such increase has previously been reported. The last years of the study (2019–2020) showed a clear decrease in the incidence of talar fractures, which to our mind due to Covid pandemia [12]. We are also unaware of any previous studies that describe the nationwide incidence of talar fractures in an adult population.

This significant increase in the incidence of talar fractures may be attributed to the improved availability of computer tomography (CT), which allows for the more accurate diagnosis of fractures, including those that are exactly positioned. As the main trauma mechanisms of talus fracture are high-energy falls and traffic accidents, CT examination is often performed in the emergency department [13].

With the recent progress in CT imaging and its improved availability, the standard diagnostics of talar fractures is now based on X-ray and CT imaging. Nowadays, CT imaging is a routine imaging modality in the emergency departments of hospitals. Therefore, the smallest suspicion of talus fracture can lead to CT imaging. This change is supported by the recent literature, since studies have revealed the advantages of CT imaging compared to X-ray in the diagnostic assessment of acute trauma [14, 15]. Moreover, the Finnish national treatment recommendations of the talus fractures have changed, so that the CT image has been included as a routine procedure in the diagnosis of the fracture.

Although the incidence of the diagnosed fractures rose significantly in the present study, the incidence of surgery remained at a steady level. The proportion of operated fractures decreased from 42.5% in 2009 to 12% in 2020. The percentage of operatively treated male and female patients started to decrease from 2009, although the number of fractures clearly increased. Between 1997 and 2008, the percentage of operatively treated fractures decreased from 42.5% (43.6% in men vs. 39.6% in women) to 12% between 2009 and 2020 (13.8% in men vs. 10.2% in women). One possible reason for this decrease is that CT was able to show exactly positioned fractures, which were then treated conservatively. Another explanation is the improved evaluation of the treatment of talar fractures and a better understanding of the conservative treatment preferences of patients [16]. Unfortunately, as there are no studies that describe the incidence of operatively treated talar fractures, there are no data to which our findings can be compared. Also, to our knowledge there are no comparative studies of operative and nonoperative treatment of talar fractures. Most studies assessing outcomes of nonoperative therapy are historical and often included management strategies such as closed reduction maneuvers (implying original displacement and a higher-grade injury) and casting [17].

The strength of this study is the nationwide register-based data which have been shown to be complete and accurate for epidemiologic purposes [8, 9, 18, 19]. Moreover, the findings of this study are not limited to scientifically active hospitals or exclusive trauma centers. In addition, Finland has a public health care system which is practically free to citizens. An additional strength of the study is the use of permanent ICD-coding and hospital registry for the whole study period, so bias in the results is not expected.

The limitation of the study is that the Finnish CRHC does not provide the laterality of the fracture, meaning that we had difficulties in separating multiple visits and hospitalizations from re-operation of the index talus or a new operation, and a fracture of the contralateral talus. Fortunately, bilateral talar fractures are very rare and only a few bilateral talar fractures have been reported to date [11]. Also, the Finnish CRHC does not provide any additional information about the fractures or computer tomographic images, so that it is impossible to divide them by any classifications. Another limitation of the study is that the data regarding the incidence of talar fracture were obtained from a CRHC, which only contains data from those patients who are treated in hospital. Therefore, fractures of the talus are sometimes missed on X-rays and treated without hospitalization and, therefore, not included in this study.

## Conclusions

In conclusion, this was the first study to describe the nationwide incidence of talar fractures and their operative treatment in Finland. The present study revealed an increased incidence of hospitalization due to talar fractures starting from the year 2009. Interestingly, the proportion of operatively treated patients started to decrease in the same year. Indeed, the percentage of operatively treated fractures decreased from 42.5 to 12% during the study period. Furthermore, the relation between operatively treated fractures in men and women almost flattened out in the second decade of the study.

## Data Availability

The datasets used and/or analysed during the current study are available from the corresponding author on reasonable request.

## References

[1] Melenevsky Y, Mackey RA, Abrahams RB, Thomson NB. Talar Fractures and Dislocations: A Radiologist’s Guide to Timely Diagnosis and Classification. Radiographics. 2015 May-Jun;35(3):765 – 79. 10.1148/rg.2015140156 25969933

[2] Kuner EH, Lindenmaier HL. Zur Behandlung Der Talusfraktur Unfallchirurgie. 1983;9:35–40. 6845530

[3] Richter M, Zech S. Behandlung Der Talusfraktur. OP J. 2007;23:104–9.

[4] Halvorson JJ, Winter SB, Teasdall RD, Scott AT. Talar neck fractures: a systematic review of the literature. J Foot Ankle Surg. 2013;52(1):56–61. 23153783 10.1053/j.jfas.2012.10.008

[5] Giordano V, Liberal BR, Rivas D, Souto DB, Yazeji H, Souza FS, Amaral NP. Surgical management of displaced talus neck fractures: single vs double approach, screw fixation alone vs screw and plating fixation–systematic review and meta-analysis. Volume 52. Injury; 2021. pp. S89–96. 10.1016/j.injury.2021.01.047 34088463

[6] Keskimaki I, Aro S. Accuracy of data on diagnosis, procedures and accidents in the Finnish Hospital Discharge Register. Int J Health Sci. 1991;2:15–21.

[7] Salmela R, Koistinen V. Is the discharge register of general hospitals complete and reliable? Sairaala. 1987;49:480–2.

[8] Mattila VM, Sillanpaa P, Iivonen T, Parkkari J, Kannus P, Pihlajamaki H. Coverage and accuracy of diagnosis of cruciate ligament injury in the Finnish National Hospital Discharge Register. Injury. 2008;39:1373–6. 18703187 10.1016/j.injury.2008.05.007

[9] Huttunen TT, Kannus P, Pihlajamaki H, Mattila VM. Pertrochanteric fracture of the femur in the Finnish National Hospital Discharge Register: validity of procedural coding, external cause for injury and diagnosis. BMC Musculoskelet Disord. 2014;15:98. 24655318 10.1186/1471-2474-15-98 PMC4026595

[10] Ponkilainen VT, Partio N, Salonen EE, Laine HJ, Maenpaa HM, Mattila VM, Haapasalo HH. Outcomes after nonoperatively treated nondisplaced lisfranc injury: a retrospective case series of 55 patients. Arch Orthop Trauma Surg. 2021;141:1311–7. 32960309 10.1007/s00402-020-03599-w PMC8295070

[11] Canale ST, Kelly FB. Fractures of the neck of the talus: long term evaluation of seventy-one cases. J Bone Joint Surg Am. 1978;60(2):143–56. 417084

[12] Nygren H, Kopra J, Kröger H, Kuitunen I, Mattila VM, Ponkilainen V, Rikkonen T, Sund R, Sirola J. The effect of COVID-19 lockdown on the incidence of emergency department visits due to injuries and the most typical fractures in 4 Finnish hospitals. Acta Orthop. 2022;93:360–6. 35257188 10.2340/17453674.2022.2252 PMC8902588

[13] Whitaker C, Turvey B, Illical E. Current concepts in Talar Neck Fracture Management. Curr Rev Musculoskelet Med. 2018;11(3):456–74. 29974334 10.1007/s12178-018-9509-9 PMC6105488

[14] Willms S, Fruson L, Buckley R. Nondisplaced talus neck fracture – operative or nonoperative care? Injury. 2023;54(4):1027–9. 36740473 10.1016/j.injury.2023.01.051

[15] Smith T, Weger K, Steenburg S. Whole body CT for trauma reduces emergency department time for patients with lower extremity fractures. Emerg Radiol. 2022;29:449–54. 35165773 10.1007/s10140-022-02030-8

[16] Avci M, Kozaci N. Comparison of X-Ray imaging and computed Tomography scan in the evaluation of knee trauma. Med (Kaunas). 2019;55(10):623. 10.3390/medicina55100623 PMC6843286 31547588

[17] Avci M, Kozaci N, Yuksel S, Etli I, Yilmaz Y. Comparison of radiography and computed tomography in emergency department evaluation of ankle trauma. Annals Med Res. 2019;26(5):867–72.

[18] Sund R. Quality of the Finnish Hospital Discharge Register: a systematic review. Scand J Public Health. 2012;40:505–15. 22899561 10.1177/1403494812456637

[19] Keskimaki I. Accuracy of data on diagnosis, procedures and accidents in the Finnish hospital register. Int J Health Sci. 1991;2:15–21.

